# Nonproportional hazards and unobserved heterogeneity in clustered survival data: When can we tell the difference?

**DOI:** 10.1002/sim.8171

**Published:** 2019-05-03

**Authors:** Theodor Adrian Balan, Hein Putter

**Affiliations:** ^1^ Medical Statistics, Department of Biomedical Data Sciences Leiden University Medical Center Leiden The Netherlands

**Keywords:** frailty, proportional hazards, unobserved heterogeneity

## Abstract

Multivariate survival data are frequently encountered in biomedical applications in the form of clustered failures (or recurrent events data). A popular way of analyzing such data is by using shared frailty models, which assume that the proportional hazards assumption holds conditional on an unobserved cluster‐specific random effect. Such models are often incorporated in more complicated joint models in survival analysis.

If the random effect distribution has finite expectation, then the conditional proportional hazards assumption does not carry over to the marginal models. It has been shown that, for univariate data, this makes it impossible to distinguish between the presence of unobserved heterogeneity (eg, due to missing covariates) and marginal nonproportional hazards. We show that time‐dependent covariate effects may falsely appear as evidence in favor of a frailty model also in the case of clustered failures or recurrent events data, when the cluster size or number of recurrent events is small. When true unobserved heterogeneity is present, the presence of nonproportional hazards leads to overestimating the frailty effect. We show that this phenomenon is somewhat mitigated as the cluster size grows.

We carry out a simulation study to assess the behavior of test statistics and estimators for frailty models in such contexts. The gamma, inverse Gaussian, and positive stable shared frailty models are contrasted using a novel software implementation for estimating semiparametric shared frailty models. Two main questions are addressed in the contexts of clustered failures and recurrent events: whether covariates with a time‐dependent effect may appear as indication of unobserved heterogeneity and whether the additional presence of unobserved heterogeneity can be detected in this case. Finally, the practical implications are illustrated in a real‐world data analysis example.

## 1 INTRODUCTION

The Cox proportional hazards model^1^ is widely used for analyzing survival data. More generally, this model may also be used for multivariate survival data, in the form of clustered failures or recurrent events.^2^ The proportional hazards assumption specifies that the ratio of the hazards (or intensities, for recurrent events) is constant in time, for any given values of the covariates. However, time‐dependent covariate effects may be observed in practice. For example, the benefit effect of a treatment may attenuate in time or a medical intervention may increase the hazard on the short term but decrease the hazard on the long term. However, if important covariates are omitted, the observed effect of modeled variables may appear time dependent, even if, at the individual level, the effect is time constant.^3^ This phenomenon is referred to as the presence unobserved heterogeneity.

Unobserved heterogeneity can be modeled explicitly via random effects, or “frailty” models. Originally introduced in the context of demographics^4^ for univariate survival data, they extend the Cox model with an individual‐specific random effect (or “frailty”). The frailty may be seen as the aggregate effect of unobserved variables on the individual hazard. Typically, the proportional hazards assumption is assumed to hold conditional on the random effect. The marginal effect of the observed covariates (ie, unconditional on the frailty) is, in general, time dependent.^5^ Such a proportional hazards frailty model is identifiable if covariates are present, under some regularity conditions.^6^

Frailty models are most often employed in a multivariate context, wherein the random effect is “shared” by a number of dependent observations.^7^, ^8^, Ch 7 Broadly, this comprises two types of data: clustered failures (where the random effect is common to individuals from the same cluster) and recurrent events (where the random effect is individual specific, but it is common to all the recurrent event episodes). In both cases, in addition to the marginal hazards, a marginal correlation structure of the dependent observation can be observed^7^: lifetimes of individuals from the same cluster are positively correlated, and so are the inter‐event times from the same individual in the case of recurrent events. This additional information means that, in theory, the time‐dependent covariate effects and the unobserved heterogeneity scenarios can be distinguished. This is not the case in univariate survival data, which can be seen as a scenario where clusters have size 1 and where individuals experience at most one event.

However, when the data are “almost univariate” (eg, cluster size is small, or only few observed recurrent events), there might be the case that no correlation structure is actually observed. For example, in twin studies, the lifetime of the second twin is always censored after birth. In this case, there is virtually no information on the correlation of the event times, but a shared frailty model is still identifiable if covariates are present.^6^ When the cluster size is small, there is indeed a confounding between the regression parameters and the dependence structure.^7^, Ch 7.2.7

In this paper, we address the following question: how “multivariate” must the data be so that shared frailty models reflect the strength of the effects of unobserved heterogeneity, rather than a possible time‐dependent effect of the observed covariates? This question is addressed through a simulation study, where we simulate time‐dependent hazard ratios and attempt to estimate different frailty models. The rest of this paper is structured as follows. In Section 2, we discuss the theoretical background of proportional hazards models and frailty models; in Section 3, we present the setup and results of the simulation study comprising a large number of scenarios; in Section 4, we review real‐life data analysis scenarios; and we present the conclusions of this study and discussion in Section 5.

## 2 MODELS

### 2.1 Proportional hazards models

In a proportional hazards model, the hazard is specified as

(1) $$\lambda (t|x)=\lambda_{0}(t)\exp(x^{⊤}\beta ),$$

where λ_0_(t) is a nonparametric “baseline” hazard, **x** is a p × 1 vector of observed covariates, and β is a p × 1 vector of unknown regression coefficients. In the case of recurrent events, (1) describes the intensity of a counting process N(t) that “counts” the number of events up to time t.^9^ For any given values of **x**, for example, **x**_1_ and **x**_2_, it may be seen that the hazard ratio does not depend on time, ie,

$$\frac{\lambda (t|x_{2})}{\lambda (t|x_{1})}=\exp\left({\left(x_{2}-x_{1}\right)}^{⊤}\beta \right).$$

If the effect of **x** on λ is time dependent, then the hazard (1) can be expressed as

(2) $$\lambda (t|x)=\lambda_{0}(t)\exp\left(x^{⊤}\beta (t)\right).$$

There are several ways of testing whether the proportional hazards assumption holds.^10^, ^11^, ^12^

In a frailty model, the hazard is specified conditional on the random effect Z, ie,

(3) $$\lambda (t|Z,x)=ZY(t)\lambda_{0}(t)\exp(x^{T}\beta ),$$

where Z is a random variable that follows a distribution with positive support. The marginal hazard corresponding to (3), obtained by integrating over the distribution of Z, is given by

(4) $$\bar{\lambda }(t|x)=E_{Z}[\lambda (t|Z,x)]$$

(5) $$=E[Z|O(t_{-})]Y(t)\exp(x^{⊤}\beta )\lambda_{0}(t),$$

where **O**(t_−_) is the event and covariate history of up to (but not including) time t, and E[Z|**O**(t_−_)] is the “posterior” expectation of Z given **O**(t_−_).

### 2.2 Marginal hazards resulting from frailty models

##### Univariate survival

Univariate survival data refers to the scenario where individuals may experience at most one event (eg, death) and they are assumed to be independent. In this case, we denote, for individual i, T_i_ as the event time, **x**_i_ as the vector of covariates, and Z_i_ as the frailty. Because the individual history at time t, O_i_(t_−_), contains only information on whether the event has happened or not,

$$E[Z_{i}|O(t_{-})]=E[Z_{i}|O_{i}(t_{-})]=E[Z_{i}|T\geq t].$$

Therefore, the marginal hazard corresponding to a covariate vector **x**_i_ is given by

$$\bar{\lambda }(t|x_{i})=E[Z_{i}|T_{i}\geq t,x_{i}]Y_{i}(t)\exp\left(x_{i}^{⊤}\beta \right)\lambda_{0}(t).$$

In general, E[Z_i_|T ≥ t,**x**_i_] is a decreasing function of time because individuals with lower frailty survive longer. Furthermore, this expectation also depends on **x**. If Z has finite variance, then the ratio of $\bar{\lambda }$ for different values of **x** is time dependent.^5^

**Figure 1:**
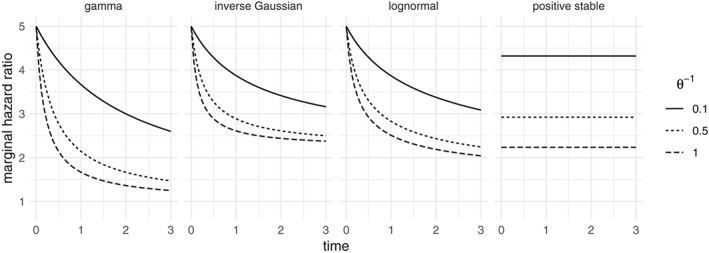
Marginal hazard ratio of survivors obtained a conditional hazard ratio of 5, for the gamma, inverse Gaussian, lognormal, and positive stable distributions, where the baseline hazard is λ_0_(t) = 1. The gamma, inverse Gaussian, and lognormal have fixed EZ = 1 and Var Z = θ ^−1^. For the positive stable, θ may still be used as a measure of association, although it is not comparable with the others. The parametrizations used here are detailed in the Appendix

In Figure 1, we show, for different frailty distributions and degrees of variability, the marginal hazard ratio between two groups of individuals that have a conditional hazard ratio of 5. For the gamma, inverse Gaussian, and lognormal distributions, the perceived attenuation of the hazard ratio reflects that the two groups become more homogeneous in time, as individuals with a higher frailty leave the data set sooner. From a practical point of view, the same decreasing hazard ratio might be explained by a true reduction in the effect of the covariate at the individual level (eg, treatment effect decreasing in time), and no unobserved heterogeneity. The last plot in Figure 1 shows the positive stable distribution. It does not have finite variance, which is one of the conditions for the identifiability result^6^ and is not identifiable from univariate survival data.^13^

##### Clustered failures

In the case of clustered failures, individuals may experince at most one event, but they are possibly correlated, with individuals nested within clusters. We denote by (*i*,*j*) individual *j* from cluster *i* and *Z*_*i*_ as the frailty that is shared by the individuals from cluster *i*. We therefore have E[*Z*_*i*_|**O**(*t*_−_)] = E[*Z*_*i*_|*O*_*i*_(*t*_−_)], where *O*_*i*_(*t*_−_) is the event history of all individuals in cluster *i*. Denote *N*_*i*_(*t*) as the number of observed events until time *t* and

$$\Lambda_{i}(t)=\overset{J_{i}}{\underset{j=1}{\sum }}\overset{t}{\underset{0}{\int }}Y_{ij}(s)\exp\left(x_{ij}^{⊤}\beta \right)\lambda_{0}(s)ds.$$

It can be shown that

(6) $$E[Z_{i}|O_{i}(t_{-})]=-\frac{L^{\left(N_{i}(t)+1\right)}(\Lambda_{i}(t))}{L^{\left(N_{i}(t)\right)}(\Lambda_{i}(t))},$$

where $L(c)=E_{Z}[\exp(-cZ)]$ is the Laplace transform of *Z* and $L^{\left(k\right)}(c)$ its *k*th derivative. The Laplace transform of common distributions is given in the Appendix.

##### Recurrent events

In the case of recurrent events, individuals are assumed to be independent, but they may experience more than one event. We denote the frailty of individual *i* as *Z*_*i*_. The event history of individual *i* is represented by a counting process *N*_*i*_ with *N*_*i*_(*t*) “counting” the number of events until time *t*. We denote *λ*_*i*_ as the intensity of *N*_*i*_, identical with expression (3). We therefore have E[*Z*_*i*_|**O**(*t*_−_)] = E[*Z*_*i*_|*O*_*i*_(*t*_−_)], where *O*_*i*_(*t*_−_) is the event history of individual *i*. For individual *i*, denote the cumulative intensity as

$$\Lambda_{i}(t)=\overset{t}{\underset{0}{\int }}Y_{i}(t)\exp\left(x_{i}^{⊤}\beta \right)\lambda_{0}(s)ds.$$

Then, (6) describes the posterior expectation of *Z*_*i*_.

It may be observed that the calculations involving recurrent events are very similar to those involving clustered failures. Further details may be found, for example, in ch 3.5 and ch 4 in the work of Cook and Lawless.^9^ Henceforth, we assume that the time scale is that of “calendar time”, where *t* is the time since the origin of the recurrent event process. Another way of modeling recurrent events is by modeling the “gap times” between events. In this case, one would specify the intensity as *λ*(*t*)≡*λ*(*t* − *B*(*t*)), where *B*(*t*) is the time since the previous event. The representation of gap times is virtually identical to the clustered failures case (with individuals playing the role of a cluster and recurrent event episodes nested within individuals) and is not treated further.

### 2.3 Nonproportional hazards or frailty?

In Figure 1, the marginal time‐dependent effect corresponding to a proportional hazards frailty model is shown for univariate survival data. If a Cox model is estimated on a data set that presents such an effect, the proportional hazards assumption will be violated. Conversely, if the frailty model that was used to simulate the data would be estimated, a positive frailty variance would be estimated. Both explanations would be equally plausible because both models lead to the same marginal effects.

In the case of clustered failures, the marginal hazard for individual *j* from cluster *i* is given by

$$\lambda_{ij}(t;x_{ij})=E[Z_{i}|O_{i}(t_{-})]\exp\left(x_{ij}^{⊤}\beta \right)\lambda_{0}(t),$$

where the expectation also depends on the covariate values of all individuals in cluster *i*. The ratio of hazards between individuals in the same cluster is time constant, whereas the ratio of hazards of individuals from different cluster is time dependent, if the two clusters have different covariate distributions and event histories.

If the frailty effect is important, then the observations within each cluster are correlated, with different frailty distributiuons leading to different correlation structures. The gamma distributions induces late dependence between event times, the positive stable induces early dependence, and the inverse Gaussian sits somewhere in the middle. This has been shown for bivariate survival data (ie, clusters of size 2).^7^

The strength of the frailty effect is usually quantified by the estimated frailty variance (or another measure of dispersion of the distribution of *Z*). In the case of univariate survival, this reflects both the importance of the unobserved heterogeneity and the strength of the violation of the proportional hazards assumption. In the case of clustered failures, a large frailty variance reflects the importance of unobserved heterogeneity that is cluster‐specific, the within‐cluster correlation structure and the between‐cluster marginal proportional hazards violation. If the cluster size is large, nonproportionality plays a smaller role because more of the within cluster correlation is observed. It becomes easier, for example, to decide whether the frailty model is more appropriate, for example, by comparing within cluster residuals with between cluster residuals.^14^

This interpretation becomes problematic when the cluster size is small. An extreme example would be that of the analysis of lifetimes of fathers and daughters in the presence of a strong risk factor.^7^ Even if all daughters would be censored and no relation between their lifetimes and the father's lifetimes can be inferred, the shared frailty model may be estimated. A similar situation occurs in the case of recurrent events when only few events are observed. In both cases, the identifiability of the frailty model relies on the result of the work of Elbers and Ridder^6^ and is likely to be influenced by potential time‐dependent covariate effects, as in the univariate case.

The main question posed by these observation is as follows: how *much* of the dependence structure must be observed so that a time‐dependent covariate effect does not appear as evidence in favor of the shared frailty model? This is studied in the following section, in the context of three scenarios: clustered failures where an observed covariate may vary within cluster (“clustered”); clustered failures where the observed covariate only varies between clusters (“clustered/common”); and recurrent events where the observed covariate varies between individuals (“recurrent”). The second scenario is also representative for the scenario where recurrent events are analyzed in the gap time scale, with gap times nested within individuals rather than individuals nested within clusters.

## 3 SIMULATION STUDY

### 3.1 General framework

We consider *x*∼Bernoulli(0.5) a binary covariate. In the simulation study, three main scenarios are analyzed. The first is that of individuals nested in clusters. The cluster size varies is chosen as 1 (univariate survival), 2, 3, 5, or 10, and *x* simulated independently for each individual. This scenario is labeled as **clustered**. The second is identical to the first scenario, with the exception that *x* is simulated independently for each cluster. This is labeled **clustered / common**. Lastly, recurrent events in calendar time are simulated,^15^ with *x* simulated independently for each individual, labeled **recurrent**. In this case, 1, 2, 3, 5, and, respectively, 10 events are simulated for each individual.

First, data are simulated from a model without unobserved heterogeneity, but with a time‐dependent effect of *x*, as described in (2). On the simulated data sets, four models are estimated: a Cox proportional intensity model and frailty models with gamma, inverse Gaussian and positive stable distributions. The Commenges‐Andersen (CA) test for heterogeneity^14^ and, for the frailty models, the likelihood ratio test for the hypothesis of no frailty are evaluated. All estimates and confidence intervals are collected. A test for the proportional hazards assumption^11^ is also evaluated to determine the degree of nonproportionality in each simulated data set. Second, the whole procedure is repeated by having data simulated also with unobserved heterogeneity in addition to the time‐dependent covariate effect. In this case, the simulated random effect follows a log‐normal distribution with mean 1 and variance 0.25.

The event times are simulated according to a Weibull distribution, which can accommodate time‐dependent covariate effects. The intensity is given by a Weibull baseline with shape and scale parameters *α* and scale *γ* and time‐dependent covariate effect $\beta_{0}+\beta_{1}\log t$, resulting in

(7) $$\lambda_{ij}(t|Z_{i};\alpha ,\gamma )=Z_{i}\alpha \gamma t^{\alpha -1}\exp\left((\beta_{0}+\beta_{1}\log t)x_{ij}\right),$$

which is again a Weibull distribution with shape *α* + *β*_1_ *x*_*ij*_ and scale $Z_{i}\alpha \gamma e^{\beta_{0}}{\left(\alpha +\beta_{1}x_{ij}\right)}^{-1}$. Both shape and scale parameters must be positive. In the case of clustered failures, this is the hazard, whereas, in the case of recurrent events, this is taken as the intensity of the recurrent events process. The baseline intensity is a decreasing function of time if *α* < 1 and decreasing for *α* > 1. For *α* = 1, the exponential distribution is obtained, where the hazard is constant.

**Figure 2:**
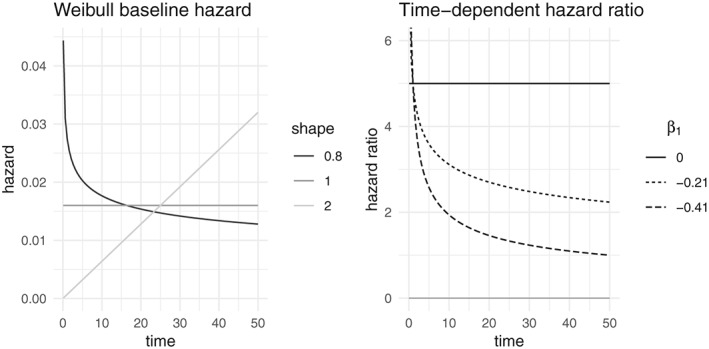
Left: Weibull baseline hazards used in the simulation, where the scale parameter is chosen so that the cumulative baseline hazard at 50 is 0.8. Right: time‐dependent hazard ratio used in the simulation and describe in Equation (7), ie, $5\exp(\beta_{1}\log t)$

The shape parameter of the Weibull distribution is taken as *α* ∈ {0.8,1,2}, corresponding to a decreasing, constant, and increasing intensity. For the clustered failures scenarios, the scale parameter is chosen so that the cumulative baseline intensity Λ_0_(50) = 0.8. The different hazard shapes are shown in Figure 2. The covariate effects are defined as in (7), with $\beta_{0}=\log(5)$, and three values for *β*_1_, denoted as $\beta_{1}^{\left(0\right)},\beta_{1}^{\left(1\right)}$, and $\beta_{1}^{\left(2\right)}$, corresponding to different degrees of time‐dependent effect. $\beta_{1}^{\left(2\right)}$ is selected so that $\beta_{0}+\beta_{1}^{\left(2\right)}\log50=0$ ); $\beta_{1}^{\left(1\right)}$ is taken as the average of 0 and $\beta_{1}^{\left(2\right)}$; and $\beta_{1}^{\left(0\right)}=0$ corresponds to the proportional hazards scenario. The corresponding hazard ratios for *α* = 0.8 are visualized in Figure 2. To keep the results comparable across scenarios, for the recurrent events with *j* events for an individual, the scale parameter is chosen so that Λ_0_(50) = 0.8*j*. Therefore, the average number of events can be compared to a cluster with *j* individuals.

Each different combination of simulation parameters leads to a different observed distribution of the event times. To keep the different scenarios comparable, an arbitrary censoring rate of 30% is imposed across all scenarios. For each combination of parameters, a large data set is simulated and the .7 quantile of the uncensored observed event time distribution is taken as censoring time for all the subsequent simulations. All calculations are performed with the R software,^16^ using the packages survival 17 and frailtyEM.^18^

### 3.2 Likelihood ratio test

The likelihood ratio test (LRT) is usually used to test the null hypothesis of *no frailty*. For the gamma and inverse Gaussian, this is equivalent to testing *H*_0_:Var [*Z*] = 0 versus *H*_*A*_:Var [*Z*] > 0. For the positive stable frailty model, for which the variance is not defined, this is equivalent to testing *H*_0_:*γ* = 0 versus *H*_*A*_:*γ* > 0, using the parametrization detailed in the Appendix. The model under *H*_0_ is equivalent to a Cox proportional intensity model assuming independent observations. It is common to approximate the distribution of the LRT statistic under *H*_0_ by a mixture distribution $\left(\chi^{2}(1)+\chi^{2}(0)\right)/2$.^19^, ^20^ This result is provided by the emfrail function in the frailtyEM R package.

##### Results: no frailty

**Figure 3:**
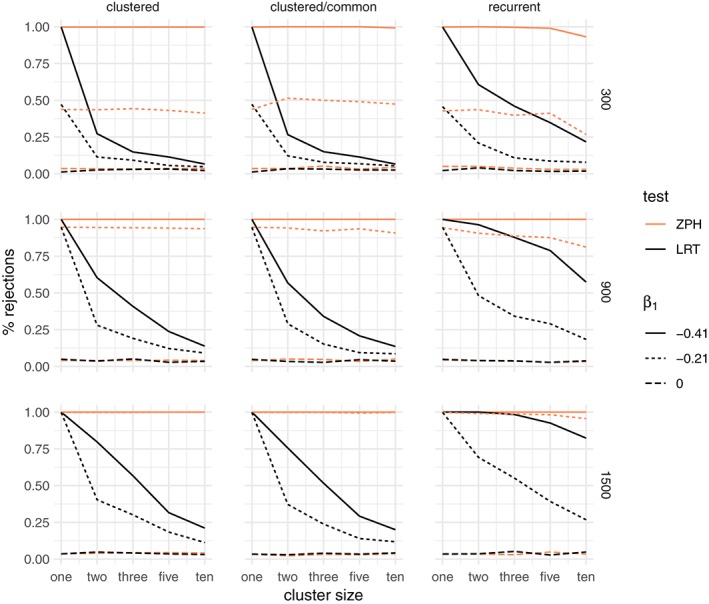
Percentage of rejections of the likelihood ratio test (LRT) between a gamma frailty model and a proportional hazard model compared to the test for nonproportional hazards (ZPH), when the data are simulated without unobserved common risk and a Weibull baseline hazard with shape α = 0.8. The rows correspond to the total sample size (300,900,1500) and the columns to the three main simulation scenarios: clustered failures, clustered failures where the observed covariate only varies between clusters, and recurrent events. β_1_ indicates the strength of the time‐dependent covariate effect [Colour figure can be viewed at wileyonlinelibrary.com]

When no frailty is included in the simulation, the percentage of rejections of *H*_0_ is shown in Figure 3, for the gamma frailty model and Weibull shape parameter is *α* = 0.8. Alongside this is the percentage of rejections of the null hypothesis of the ZPH test for proportionality.^11^

When the data are indeed simulated with proportional hazards (*β*_1_ = 0), the percentage of rejections for both tests is close to the nominal alpha level of 5% across all scenarios, regardless of cluster size. When the hazards are not proportional (*β*_1_ < 0), the percentage of rejections grows with total sample size. For larger cluster sizes, the LRT shows a decreasing number of false positives. In particular, for smaller clusters, there is a visibly large proportion of rejections, even when the time‐dependent covariate effect is moderate. The rate of rejections of the ZPH test does not appear to be strongly influenced by the cluster size. Whether the covariate varies within the cluster (the “clustered” case) or only between clusters (“clustered/common” case) does not make a practical difference. These observations carry over also for the recurrent events. The conclusion is that the time‐dependent covariate effect alone may appear as evidence in favor of the gamma frailty model, unless the cluster size is moderate to large. The results for the other Weibull shape parameters are shown in the supplementary material (Figures S1 and S2). The results for the inverse Gaussian frailty are very similar to those of the gamma frailty and can be found in the supplementary material (Figures S9, S10, and S11).

**Figure 4:**
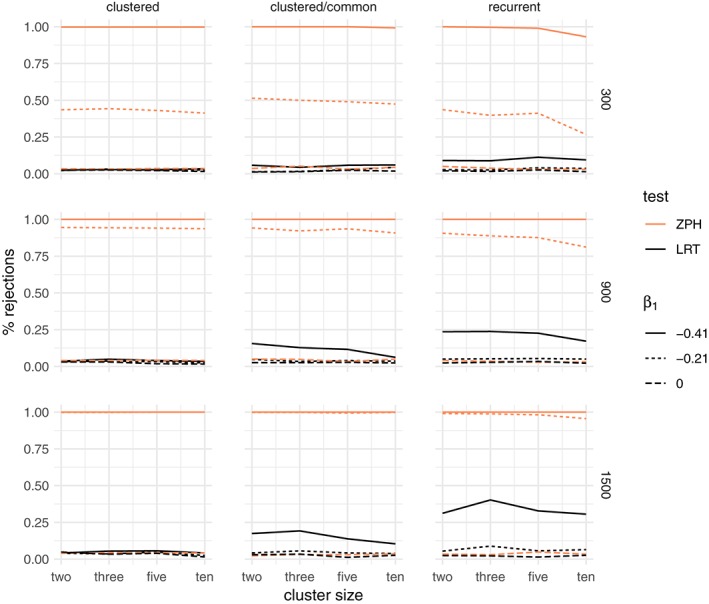
Percentage of rejections of the likelihood ratio test (LRT) between a positive stable frailty model and a proportional hazard model compared to the test for nonproportional hazards (ZPH), when the data are simulated without unobserved common risk and an increasing Weibull baseline hazard with shape α = 0.8. The rows correspond to the total sample size (300,900,1500) and the columns to the three main simulation scenarios: clustered failures, clustered failures where the observed covariate only varies between clusters, and recurrent events. β_1_ indicates the strength of the time‐dependent covariate effect [Colour figure can be viewed at wileyonlinelibrary.com]

For the positive stable distribution, the corresponding results are shown in Figure 4. In the case of clustered events, the LRT shows around 5% rejections regardless of the degree of nonproportionality. However, when the covariate does not vary within cluster or in the case of recurrent events, where the covariate is constant for each individual, the large amount of nonproportionality may still be somewhat confounded with unobserved heterogeneity. This is explained by the fact that, in these cases, there is virtually no observed within‐cluster heterogeneity. Therefore, the differences explained by *x* are essentially confounded with the differences that may be explained by cluster‐specific unobserved heterogeneity. The conclusion is that the positive stable distribution is not affected by the departures from proportionality as long as there is some within‐cluster variation of the observed covariates. The results for the other Weibull shape parameters are shown in the supplementary material (Figures S5 and S6).

##### Results: frailty

When the data are simulated as before, but also with unobserved heterogeneity, the percentage of rejections of the LRT is larger, as expected, and the ZPH test rejects the null hypothesis more than 5% of the time. This is due to the fact that marginal nonproportionality arises both from the time‐dependent covariate effect and from the frailty effect.

**Figure 5:**
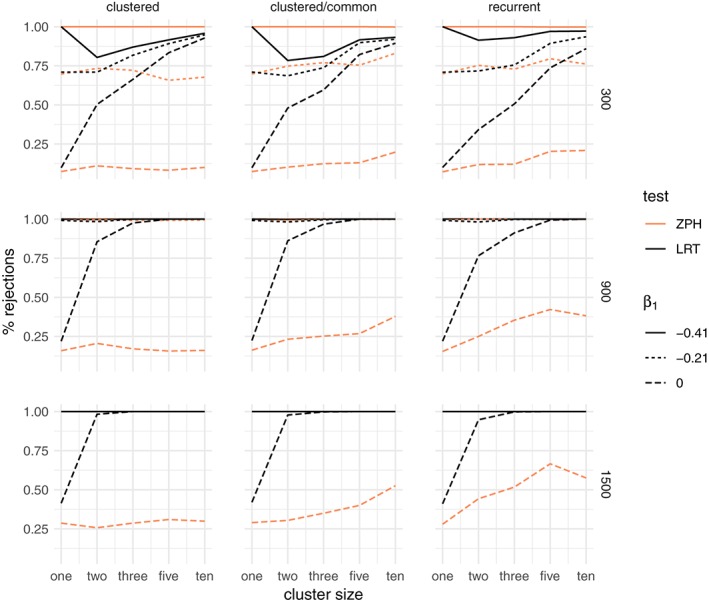
Percentage of rejections of the likelihood ratio test (LRT) between a gamma frailty model and a proportional hazard model compared to the test for nonproportional hazards (ZPH), when the data are simulated with an unobserved common risk following a lognormal distribution with expectation 1 and variance 0.25 and an increasing Weibull baseline hazard with shape α = 0.8. The rows correspond to the total sample size (300,900,1500) and the columns to the three main simulation scenarios: clustered failures, clustered failures where the observed covariate only varies between clusters, and recurrent events. β_1_ indicates the strength of the time‐dependent covariate effect [Colour figure can be viewed at wileyonlinelibrary.com]

The results for the gamma frailty model are shown in Figure 5. Even under conditional proportional hazards (*β*_1_ = 0), the LRT rejects the null hypothesis more than 5% of the times. In the scenarios where the covariate does not vary between clusters (including the recurrent events), the power of the ZPH test increases with cluster size. Therefore, presence of such a time‐dependent covariate effect in addition to unobserved heterogeneity increases the power of the LRT.

**Figure 6:**
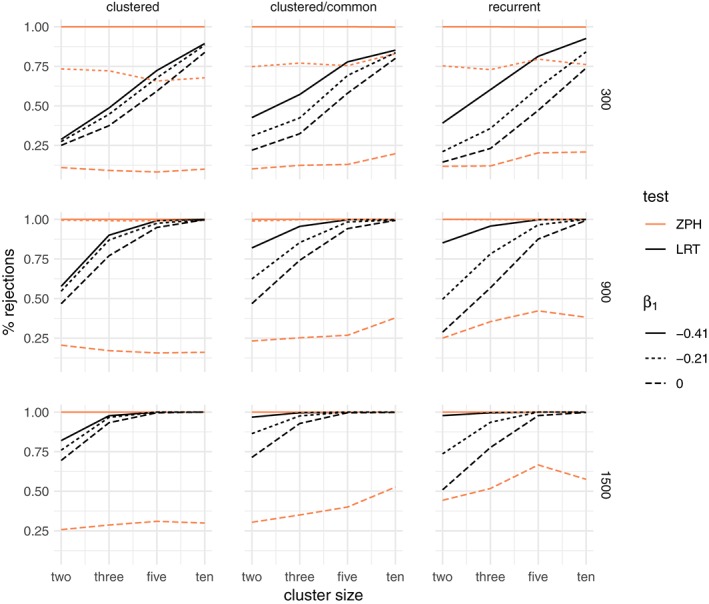
Percentage of rejections of the likelihood ratio test (LRT) between a positive stable frailty model and a proportional hazard model compared to the test for nonproportional hazards (ZPH), when the data are simulated with an unobserved common risk following a lognormal distribution with expectation 1 and variance 0.25 and an increasing Weibull baseline hazard with shape α = 0.8. The rows correspond to the total sample size (300,900,1500) and the columns to the three main simulation scenarios: clustered failures, clustered failures where the observed covariate only varies between clusters, and recurrent events. β_1_ indicates the strength of the time‐dependent covariate effect [Colour figure can be viewed at wileyonlinelibrary.com]

The results for the positive stable frailty model are shown in Figure 6. In this case, a visible effect is that of the degree of nonproportionality. A stronger time‐dependent effect of the covariate leads to a substantially larger proportion of rejections.

Although the data were simulated with unobserved heterogeneity, the difference in the rate of rejections when *β*_1_ < 0 as compared to *β*_1_ = 0 may be regarded as *rejecting the null hypothesis for the wrong reasons*.

In conclusion, time‐dependent covariate effects may appear as evidence in favor of frailty models, even if unobserved heterogeneity does not actually exist. If that exists, then the nonproportionality of the covariate effect may lead to overestimating the evidence in favor of the frailty model. The results for other shapes of the baseline hazard are shown in the supplementary material (Figures S3 and S4 for gamma frailty, S7 and S8 for the positive stable frailty). Similar conclusions apply in those cases as well, although the percentage of rejections is the largest for the decreasing baseline hazard (as shown here). This is explained in part by the fact that, with a decreasing hazard, events occur earlier on in the follow‐up, leading to earlier censoring. The resulting smaller window of observation makes the *observed* time‐dependent hazard ratio more compatible with the one predicted by the frailty models shown in Figure 1. The results for the inverse Gaussian frailty model are again very similar to the gamma frailty and are shown in the supplementary material (Figures S12, S13, and S14).

### 3.3 Commenges‐Andersen test

The CA test for heterogeneity shows in general the same behavior as the LRT from the gamma frailty or inverse Gaussian frailty models, albeit with slightly fewer rejections. This is not surprising because it is a score test, which are generally less powerful than LRT's. For example, in Table 1, the CA, LRT, and ZPH tests are shown side‐by‐side for varying cluster sizes for total sample size of 300 and Weibull shape parameter 1.

**Table 1:** Percentage of rejection of the null hypothesis for the Commenges‐Andersen, ZPH, and likelihood ratio tests (LRTs) for gamma (GA), inverse Gaussian (IG), and positive stable (PS) frailty models, for different cluster sizes (n). $\sigma_{1}^{2}$ is the variance of the lognormal frailty used in the simulation and β_1_ represents the strength of the time‐dependent part of the covariate effect as in Equation (7). The results are shown for a total sample size of 300 and Weibull shape parameter α = 1

		Clustered				Clustered / Common				Recurrent			
	Test	*n* = **2**	*n* = **3**	*n* = **5**	*n* = **10**	*n* = **2**	*n* = **3**	*n* = **5**	*n* = **10**	*n* = **2**	*n* = **3**	*n* = **5**	*n* = **10**
*σ* ^2^ = 0
*β*_1_ = 0	CA	0.020	0.046	0.048	0.044	0.062	0.064	0.042	0.060	0.060	0.034	0.036	0.038
	ZPH	0.034	0.032	0.036	0.036	0.036	0.052	0.032	0.044	0.050	0.038	0.030	0.030
	LRT (GA)	0.026	0.030	0.032	0.022	0.034	0.032	0.026	0.026	0.040	0.022	0.016	0.018
	LRT (IG)	0.026	0.028	0.032	0.022	0.032	0.032	0.028	0.026	0.038	0.026	0.022	0.020
	LRT (PS)	0.024	0.026	0.022	0.016	0.012	0.014	0.024	0.018	0.020	0.016	0.026	0.014
*β*_1_ = −0.21	CA	0.050	0.054	0.052	0.056	0.074	0.044	0.062	0.054	0.122	0.068	0.064	0.074
	ZPH	0.327	0.329	0.315	0.293	0.382	0.328	0.358	0.294	0.301	0.293	0.285	0.173
	LRT (GA)	0.078	0.066	0.042	0.044	0.084	0.048	0.052	0.038	0.155	0.082	0.066	0.070
	LRT (IG)	0.080	0.070	0.044	0.048	0.090	0.044	0.052	0.038	0.145	0.074	0.066	0.064
	LRT (PS)	0.024	0.032	0.026	0.024	0.016	0.018	0.028	0.038	0.026	0.022	0.032	0.028
*β*_1_ = −0.41	CA	0.078	0.066	0.062	0.060	0.100	0.064	0.068	0.050	0.263	0.153	0.127	0.094
	ZPH	0.952	0.954	0.948	0.942	0.960	0.964	0.952	0.942	0.956	0.920	0.924	0.857
	LRT (GA)	0.120	0.090	0.062	0.052	0.122	0.076	0.062	0.044	0.313	0.197	0.151	0.096
	LRT (IG)	0.110	0.092	0.062	0.056	0.118	0.070	0.066	0.046	0.283	0.201	0.159	0.106
	LRT (PS)	0.026	0.028	0.028	0.030	0.046	0.032	0.042	0.048	0.054	0.058	0.062	0.068
*σ* ^2^ = 0.25
*β*_1_ = 0	CA	0.415	0.565	0.770	0.910	0.404	0.526	0.772	0.876	0.309	0.460	0.691	0.837
	ZPH	0.110	0.092	0.082	0.100	0.102	0.124	0.130	0.198	0.118	0.120	0.203	0.209
	LRT (GA)	0.503	0.663	0.834	0.928	0.480	0.596	0.822	0.894	0.341	0.506	0.737	0.859
	LRT (IG)	0.511	0.679	0.842	0.932	0.492	0.604	0.832	0.902	0.359	0.512	0.737	0.867
	LRT (PS)	0.251	0.375	0.593	0.838	0.220	0.324	0.580	0.800	0.145	0.231	0.472	0.739
*β*_1_ = −0.21	CA	0.591	0.693	0.836	0.938	0.570	0.644	0.868	0.894	0.530	0.629	0.835	0.916
	ZPH	0.513	0.519	0.489	0.527	0.576	0.576	0.622	0.668	0.600	0.590	0.663	0.665
	LRT (GA)	0.667	0.776	0.880	0.952	0.640	0.718	0.886	0.912	0.590	0.669	0.855	0.918
	LRT (IG)	0.665	0.776	0.890	0.948	0.642	0.716	0.890	0.920	0.588	0.677	0.867	0.924
	LRT (PS)	0.273	0.429	0.669	0.874	0.286	0.396	0.674	0.818	0.209	0.323	0.580	0.827
*β*_1_ = −0.41	CA	0.591	0.703	0.862	0.934	0.570	0.664	0.848	0.906	0.657	0.719	0.880	0.938
	ZPH	0.984	0.976	0.980	0.978	0.998	0.986	0.990	0.990	0.996	0.984	0.980	0.988
	LRT (GA)	0.667	0.776	0.888	0.940	0.638	0.724	0.884	0.920	0.715	0.767	0.906	0.944
	LRT (IG)	0.669	0.782	0.888	0.944	0.640	0.724	0.890	0.924	0.727	0.779	0.906	0.944
	LRT (PS)	0.255	0.451	0.683	0.876	0.370	0.488	0.712	0.832	0.295	0.452	0.711	0.880

### 3.4 Estimated frailty variance

**Figure 7:**
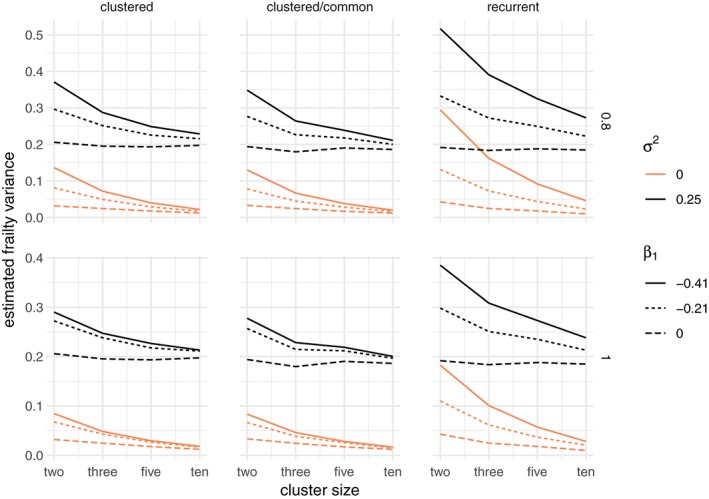
Estimated frailty variance for a gamma frailty model, when the data are simulated with an unobserved common risk following a lognormal distribution with expectation 1 and variance σ ^2^ ∈ {0,0.25} and a total sample size of 300. The rows correspond to the Weibull baseline shape parameter, increasing for α = 0.8 and constant for α = 1. The columns correspond to the three main simulation scenarios: clustered failures, clustered failures where the observed covariate only varies between clusters, and recurrent events. β_1_ indicates the strength of the time‐dependent covariate effect [Colour figure can be viewed at wileyonlinelibrary.com]

In the case of the gamma frailty, the estimated frailty variance is often considered an indication of the strength of the frailty effect. For the univariate case, these estimates were very large under all scenarios of nonproportionality. In the data sets simulated without frailty, the estimates decrease toward 0 with increasing cluster size and are not influenced by the total sample size across all scenarios, whereas they are larger with increased departure from proportional hazards. When data sets were simulated with frailty, a similar phenomenon is observed, although the estimates approach a value close to 0.25, which is the variance of the lognormal simulated frailty. This is illustrated, for a total sample of 900 and for the decreasing and constant hazard shapes in Figure 7.

**Figure 8:**
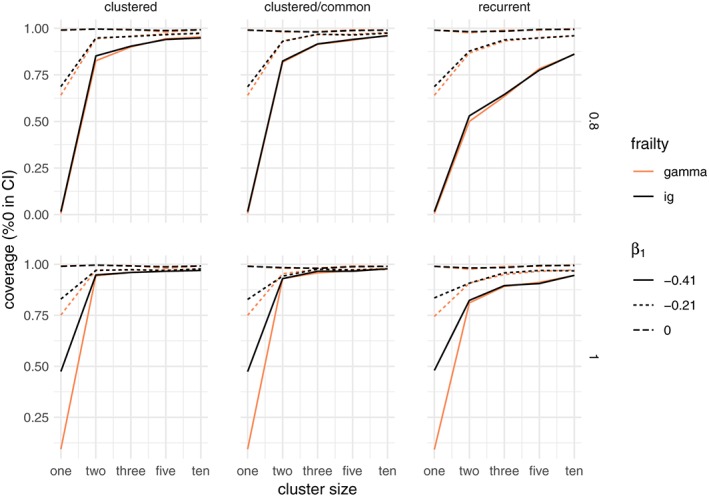
Coverage of the likelihood‐based confidence interval for the gamma frailty variance for the gamma and inverse Gaussian distributions, when the data are simulated with no unobserved heterogeneity (true variance is 0) and a total sample size of 300. The rows correspond to the Weibull baseline shape parameter, increasing for α = 0.8 and constant for α = 1. The columns correspond to the three main simulation scenarios: clustered failures, clustered failures where the observed covariate only varies between clusters, and recurrent events. β_1_ indicates the strength of the time‐dependent covariate effect [Colour figure can be viewed at wileyonlinelibrary.com]

The coverage of the frailty variance estimates can be analyzed with the likelihood‐based confidence intervals implemented in the frailtyEM package. There is a 1‐1 correspondence between the lower bound of this confidence interval being 0 and the rejection of the LRT null hypothesis. As expected, in the univariate case, the coverage is almost 0 under nonproportionality, and it improves with larger cluster size. The degree of departure from proportionality, as in the case of the LRT, plays a large role in determining whether the confidence interval of the estimated frailty variance includes 0 or not. For a total sample of 900 and for the decreasing and constant hazard, this is shown in Figure 8.

### 3.5 Cumulative hazard

As shown in Section 2, the observed hazard ratio of the groups defined by the values of *x* can be determined by integrating out the frailty. In the case of no frailty and *β*_1_ = 0, all methods estimate roughly the same cumulative marginal hazard at the end of follow‐up. If *β*_1_ < 0, the models also act similarly: the fitted cumulative hazard for *x* = 0 is larger and that for *x* = 1 is lower, resulting in the shrinkage phenomenon shown in Figure 1.

In the case when a frailty effect is also included in the simulation, the gamma and inverse Gaussian show similar results. The positive stable distribution is slightly closer to the marginal Cox model because both models specify a marginal model where the hazards are proportional.

### 3.6 Bivariate dependence

**Figure 9:**
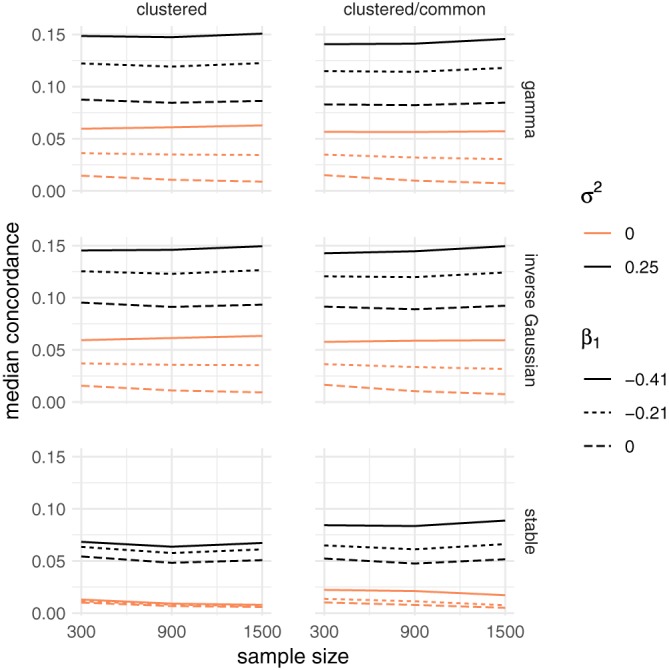
Average median concordance for the bivariate scenario, when the data are simulated with an unobserved common risk following a lognormal distribution with expectation 1 and variance σ ^2^ ∈ {0,0.25}. The rows correspond to the gamma, inverse Gaussian, and positive stable frailty models. The columns correspond to the clustered failures and clustered failures where the observed covariate only varies between clusters scenarios. β_1_ indicates the strength of the time‐dependent covariate effect [Colour figure can be viewed at wileyonlinelibrary.com]

Several bivariate dependence measures have been proposed in the literature.^7^ For clusters of size 2, the median concordance is defined as

(8) $$\kappa =E\left[\left(T_{1}-\text{median}\;(T_{1})\right)\left(T_{2}-\text{median}\;(T_{2})\right)\right],$$

or equivalently as *κ* = 2*p* − 1, where *p* is the probability that both event times are on the same side of the median event time. Closed‐form formulas are available for the median concordance for the gamma, inverse Gaussian, and positive stable distributions^7^ and are detailed in the Appendix. Results are shown in Figure 9. For all frailty models, it can be seen that stronger time‐dependent covariate effects may appear as stronger dependence. The impact of sample size on these estimates is negligible. For the gamma and inverse Gaussian distribution, it does not matter whether the covariate varies within clusters or not. The positive stable distribution estimates lower values of the median concordance. A similar phenomenon was observed in Figure 4, where the likelihood ratio test would be rejected in the clustered/common case, but not in the clustered case.

## 4 APPLICATION

### 4.1 Kidney cathether insertions

The kidney catheter data^21^ have often been used to illustrate the use of frailty models for recurrent events. Recurrent times to infection for 38 patients that use portable dialysis equipment were recorded. A gap time may be censored when the catheter is removed for a reason other than infection. At most, two gap times are included for each individual. For 23 patients, there were two observed events, for 12 patients, there was one observed event and one censored, whereas, for 3 patients, both gap times were censored. The observed covariates consist of age, sex, and disease type (four‐level categorical variable).

The data set is included in the survival package^17^ in the R statistical software.^16^ A gamma frailty model without any covariates leads to an estimated frailty variance of 0.177 with a 95% CI [0,0.985], which is not significant (*p* = 0.259 for the LRT, *p* = 0.22 for C‐A). While the addition of age does not impact the model fit in an important way, the addition of sex leads to an estimated frailty variance of 0.388 with a 95% CI [0.04,1.01], which is significant (*p* = 0.012 for the LRT, *p* = 0.002 for the CA test). The effect of sex is also highly significant, with *β* = −1.55 (0.49). With the removal of an outlier (a male with very long observed gap times), the evidence in favor of the frailty model disappears,^3^(Ch 9.5) where the authors note that *with this subject in the model, it is a toss‐up whether the disease or the frailty term will be credited with “significance”*. Nevertheless, it is remarkable that the frailty variance estimate increases with the addition of a covariate, which, in principle, should account for part of the heterogeneity in the data.

**Figure 10:**
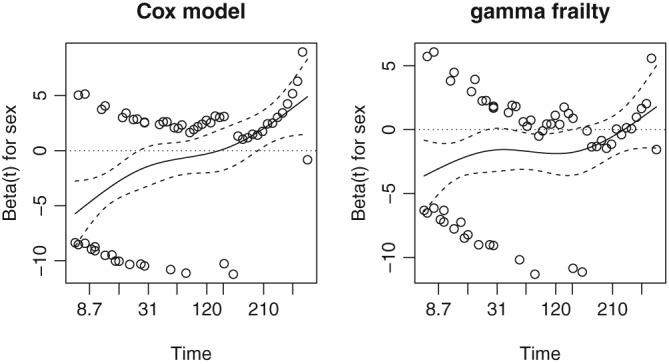
Plot of the Schoenfeld residuals for sex from a Cox marginal model and a gamma frailty model estimated on the kidney catheter insertions data

A Cox proportional hazards no‐frailty model including age and sex as covariates show a reduced effect of sex with *β* = −0.82 (0.48), ie, not significant. Furthermore, the effect of sex is highly nonproportional (*p* < 0.01). Plots of the Schoenfeld residuals from this model and a model with the logarithm of the posterior gamma frailty expectations included as an offset are shown in Figure 10. The departure from proportionality is represented by the departure of the fitted line from a horizontal line. It can be seen that the gamma frailty model “fixes” this by taking the marginal time‐dependent effect as evidence for the effect of unobserved heterogeneity.

An ad‐hoc way of modeling time‐dependent effects is by fitting an extended model where an interaction between sex and time is also included. The interaction is highly significant with *β* = −0.016 (0.002), whereas the main effect of sex is of an opposite sign *β* = 0.88(0.47). This implies a decreasing effect of sex with *β*(*t*) = 0.88 − 0.016 *t*. At the median catheter survival time, the effect of sex is already negative with *β*(78) = −0.37. Since the effect of the usual frailty distributions leads to an attenuation of the marginal hazard ratio but not to a change of signs in *β*(*t*) (as can be seen, for example, in Figure 1), it is likely that there is a time‐dependent effect of sex acting at the individual level.

A shared frailty model using a positive stable distribution for the random effect does not show a significant frailty. It was seen in the previous section that this distribution is less susceptible to rejecting the null hypothesis of no frailty because of time‐dependent covariate effects.

Therefore, two competing explanations are plausible. The first is that there is unobserved heterogeneity and a time‐constant effect of sex that appears time‐dependent (as it does with the marginal model implied by the gamma frailty). The second is that the apparent unobserved heterogeneity is an artifact induced by a time‐dependent effect of sex. Deciding between these two on the basis of these results alone is a difficult matter. This is in line with the explanation that nonproportional hazard effects and unobserved heterogeneity are confounded when the cluster size is small, as was shown in Section 3. Finally, we note that, if the third variable (disease type) is included in the model, the evidence in favor of the frailty vanishes.

## 5 CONCLUSION

In univariate survival data, it is well known that a proportional hazards frailty model and a nonproportional hazards model (with a certain type of departure from proportionality) cannot be distinguished on the basis of the data alone. We have studied how this problem extends to correlated survival data, such as clustered failures or recurrent events. The novelty of this paper is that the confounding effect between marginal covariate effects and cluster effects was studied for different cluster sizes, and reasonable rates of false rejections are obtained only when the cluster size is large (eg, 10 or more observations). Furthermore, the shape of the baseline hazard was shown to have a strong effect, with hazards that are large early on in the follow‐up more likely to be influenced by the time‐dependent effect of the covariates.

Although the simulation study in Section 3 aimed to cover a large number of scenarios, only a particular type of covariate effect was considered. In practice, this effect may be very different according to the true mechanism that generates the data. Nevertheless, this consideration should play an essential role in deciding whether the frailty model is plausible or not. We found that the conclusions presented in Section 3 extend to a large number of scenarios, including a similar simulation study carried out with a Gompertz baseline hazard. The Gompertz distribution with a time‐dependent effect of the type *β*_0_ + *β*_1_ *t* is easily simulated, as the resulting event time distribution is also Gompertz. In this paper, only the results based on a Weibull baseline hazard were shown because the Gompertz hazards are increasing, whereas the Weibull can be both increasing or decreasing.

The test for the proportional hazards assumption employed here is based on the work of Grambsch and Therneau,^11^ and the default settings of its implementation in the **survival** package in R was employed.^17^ The rationale for this is that it is likely that this is the most easily accessible test due to its inclusion with standard statistical software. There are however other ways of assessing the proportional hazards assumption that were not considered here.^10^, ^12^

The frailty models used to model unobserved heterogeneity in this paper are essentially random intercept models. Another possible extension would be to consider correlated frailty models.^22^, ^23^ In that case, the individuals from the same cluster would only share part of the frailty, with another part being individual specific. The simulation scenarios in Section 3 can be seen as a particular case, when the individual‐specific part is zero. Therefore, the conclusions of the simulation study would still apply: nonproportional hazards may still provide evidence for a frailty model. However, the extent to which the individual‐specific or cluster‐specific parts of the frailty are influenced is still a matter for future research.

A scenario worth further investigation is that when the frailty is present and a covariate has an increasingly protective effect. This would translate, in the terms of Equation (7), as having *β*_1_ > 0 and Var [*Z*] > 0. This may be seen as the time‐dependent covariate effect offsetting the shrinking of the hazard ratio seen in Figure 1. In particular, if time‐dependent covariate effects are present in addition to unobserved heterogeneity, the two effects are likely to prove difficult to disentangle.

All fitted models aim to accommodate the observable quantities according to different assumptions. The marginal hazards and marginal hazard ratios are somewhat more interpretable, as they “stick to this world”.^24^ Identifying the nature of what leads to the observable effects involves an additional number of assumptions that should be carefully considered in the problem being analyzed.

## Supporting information

SIM_8171‐Supp‐0001‐supplementary.pdf

## 1

The frailty distributions described used in this paper are all members of the power‐variance‐function (PVF) family of distributions.^7^ We describe their parametrizations by denoting *γ* as the scale parameter and *α* as the shape parameter. The Laplace transform of a PVF distributions is given by

$$L_{Z}(c)=\exp(-\alpha \psi (c;\gamma )),$$

with *ψ* a real valued function. The median concordance *κ*, defined in Equation (8), can be calculated as

$$\kappa =4L\left(L^{-1}(1/2)+L^{-1}(1/2)\right)-1.$$

The Gamma(*α*,*γ*) distribution is described by the Laplace transform

$$L_{Z}(c)={\left(\frac{\gamma }{\gamma +c}\right)}^{\alpha }.$$

This is scaled by setting E*Z* = 1 and variance *θ* ^−1^ by *γ* = *α* = *θ*.

The inverse Gaussian distribution IG(*α*,*γ*) is described by the Laplace transform

$$L_{Z}(c)=\exp\left[-\alpha \left\{{\left(\frac{\gamma +c}{\gamma }\right)}^{1/2}-1\right\}\right].$$

This is scaled by setting E*Z* = 1 and variance *θ* ^−1^ by *γ* = *θ*/2 and *α* = *θ*.

The positive stable distribution PS(*α*,*γ*) with *γ* ∈ [0,1] is described by the Laplace transform

$$L_{Z}(c)=\exp\left(-\alpha c^{\gamma }\right).$$

This is scaled with $\gamma =\frac{\theta }{\theta +1}$ and *α* = 1. The expectation is infinite and the variance is not defined. Nevertheless, with *θ* = *∞* (*γ* = 1), the case of no association is obtained and the distribution only has mass at 1, whereas smaller values of *θ* indicate higher degrees of association.

For all the distributions earlier, the LRT tests the null hypothesis of *H*_0_:*θ* = *∞*, equivalent to no variability in the frailty distribution.

The lognormal distribution *LN*(*μ*,*σ* ^2^) is usually parametrized on the log scale, ie, ElogZ=μ and $\mathrm{Var}\;\log Z=\sigma^{2}$. In Section 3, the frailty was simulated by setting E*Z* = 1 and Var *Z* = *θ* ^−1^, which is LN(−1/2log(θ+1),log(θ+1)). The Laplace transform is not available in closed form. However, for *Z* a *LN*(*μ*,*σ* ^2^), a common approximation is

$$L_{Z}(c)={\left(1+W(e^{\mu }\sigma^{2}c)\right)}^{-1/2}\exp\left(-\frac{W^{2}(e^{\mu }\sigma^{2}c)+2W(e^{\mu }\sigma^{2}c)}{2\sigma^{2}}\right),$$

where *W*(*x*) is the Lambert *W* function.^25^
