# Supplementary material for: Nonproportional hazards and unobserved heterogeneity in clustered survival data: When can we tell the difference?
Source: Stat Med. 2019 May 3;38(18):3405–20. doi: 10.1002/sim.8171 (PMC6619282; doi:10.1002/sim.8171)
Supplement: Supplementary file 1 — SIM_8171‐Supp‐0001‐supplementary.pdf [file SIM-38-3405-s001.pdf]

Non-proportional hazards and unobserved heterogeneity in clustered survival data: When can we tell the difference?

Supplementary Material

T. A. Balan & H. Putter

1 Likelihood Ratio Test - Gamma

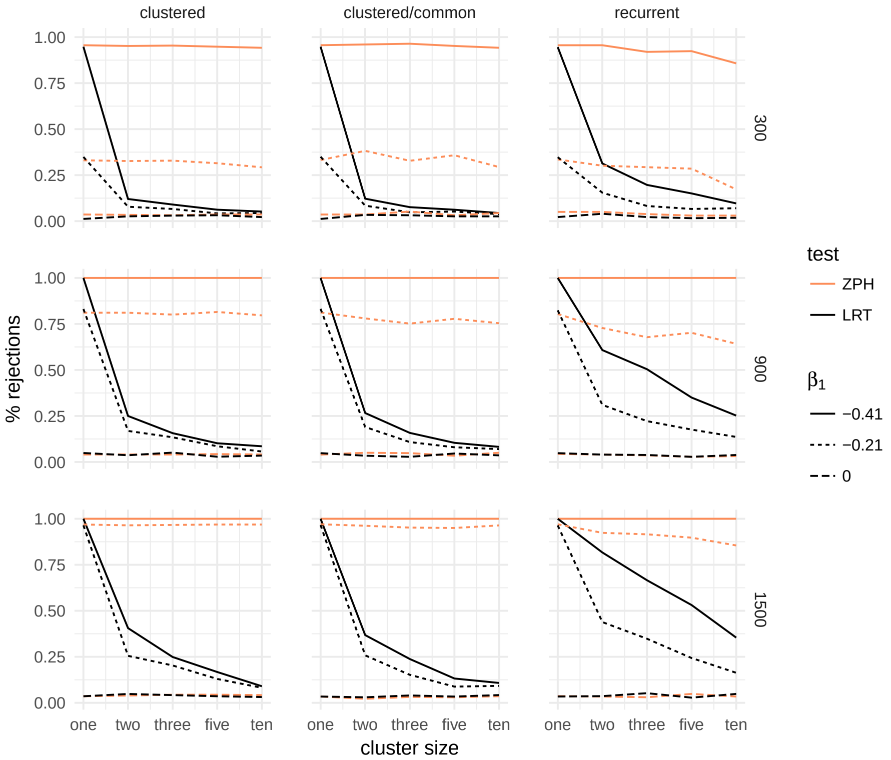

Figure S1: Percentage of rejections of the likelihood ratio test (LRT) between a gamma frailty model and a proportional hazard model compared to the test for non-proportional hazards (ZPH), when the data are simulated without unobserved common risk and an increasing Weibull baseline hazard with shape $\alpha = 1$. The rows correspond to the total sample size $(300, 900, 1500)$ and the columns to the three main simulation scenarios: clustered failures, clustered failures where the observed covariate only varies between clusters, and recurrent events. β_1 indicates the strength of the time-dependent covariate effect.

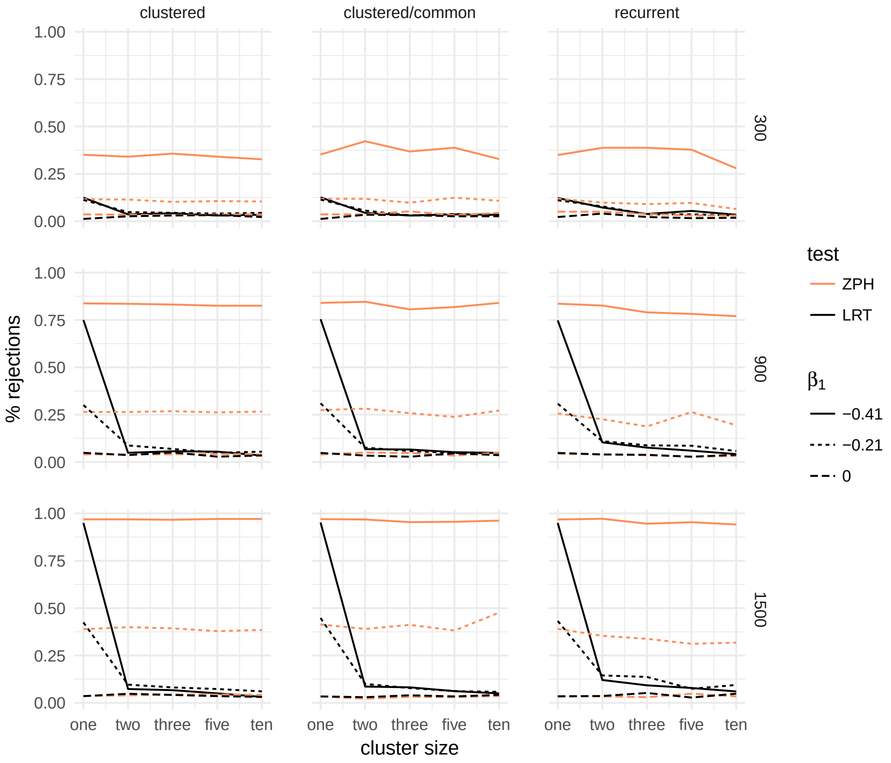

Figure S2: Percentage of rejections of the likelihood ratio test (LRT) between a gamma frailty model and a proportional hazard model compared to the test for non-proportional hazards (ZPH), when the data are simulated without unobserved common risk and an increasing Weibull baseline hazard with shape $\alpha = 2$. The rows correspond to the total sample size $(300, 900, 1500)$ and the columns to the three main simulation scenarios: clustered failures, clustered failures where the observed covariate only varies between clusters, and recurrent events. β_1 indicates the strength of the time-dependent covariate effect.

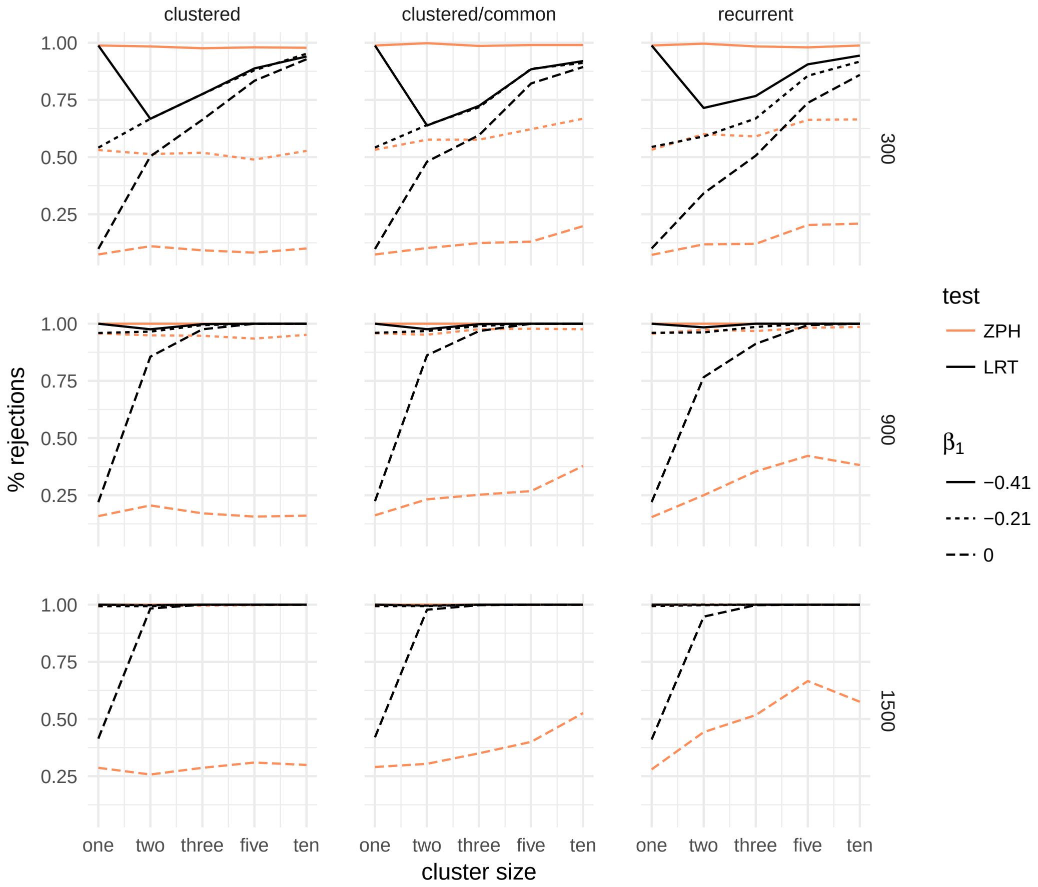

Figure S3: Percentage of rejections of the likelihood ratio test (LRT) between a gamma frailty model and a proportional hazard model compared to the test for non-proportional hazards (ZPH), when the data are simulated with an unobserved common risk following a log-normal distribution with expectation 1 and variance 0.25 and an increasing Weibull baseline hazard with shape $\alpha = 1$. The rows correspond to the total sample size $(300, 900, 1500)$ and the columns to the three main simulation scenarios: clustered failures, clustered failures where the observed covariate only varies between clusters, and recurrent events. β_1 indicates the strength of the time-dependent covariate effect.

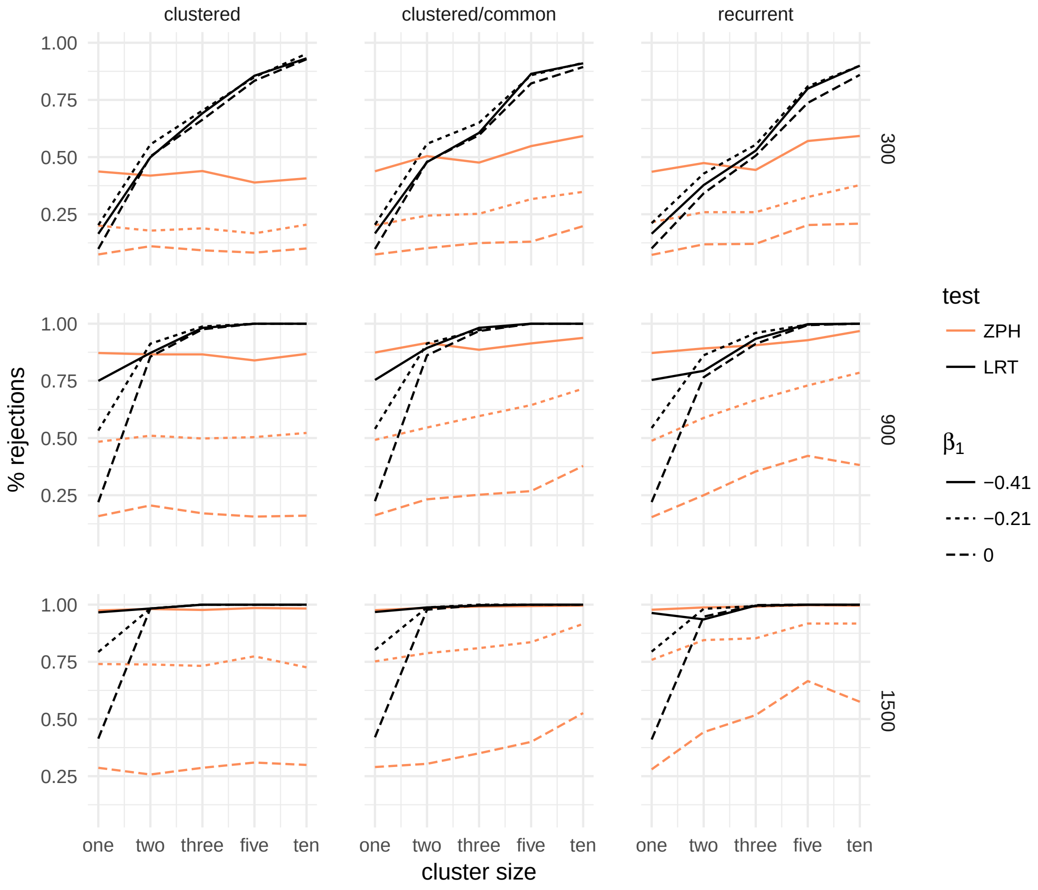

Figure S4: Percentage of rejections of the likelihood ratio test (LRT) between a gamma frailty model and a proportional hazard model compared to the test for non-proportional hazards (ZPH), when the data are simulated with an unobserved common risk following a log-normal distribution with expectation 1 and variance 0.25 and an increasing Weibull baseline hazard with shape $\alpha = 2$. The rows correspond to the total sample size $(300, 900, 1500)$ and the columns to the three main simulation scenarios: clustered failures, clustered failures where the observed covariate only varies between clusters, and recurrent events. β_1 indicates the strength of the time-dependent covariate effect.

Likelihood Ratio Test - Positive stable

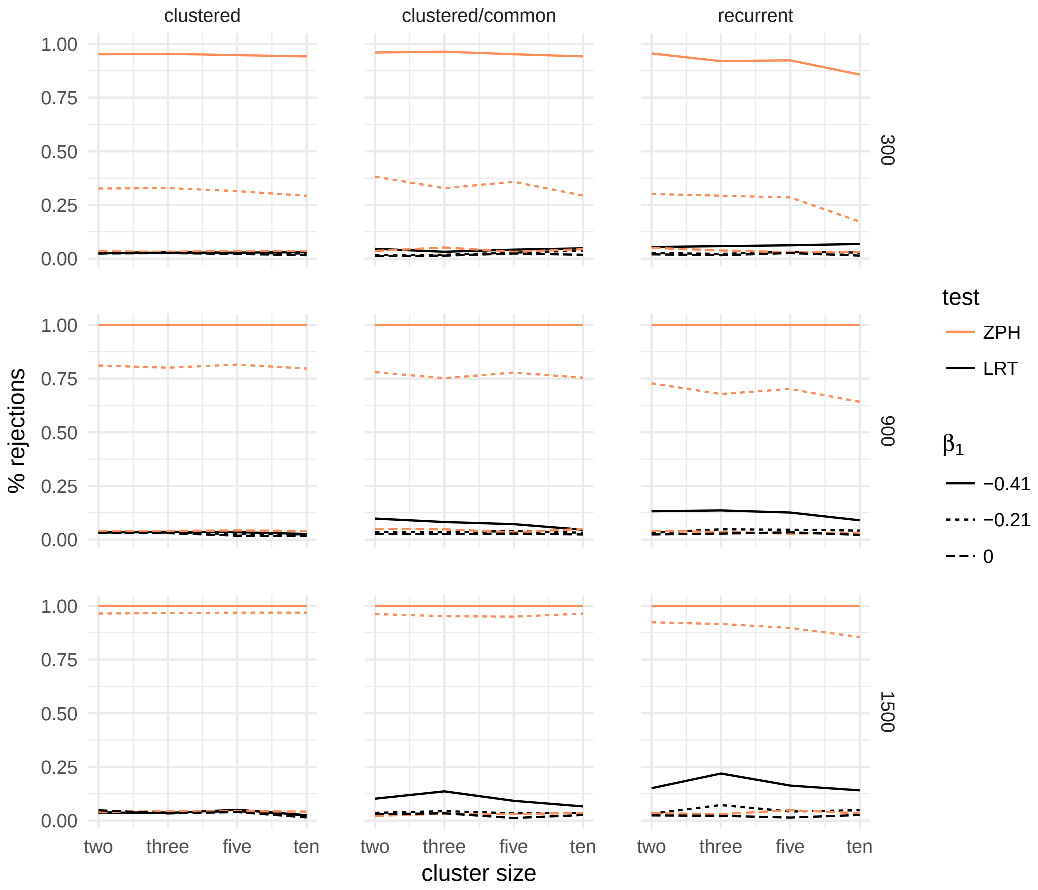

Figure S5: Percentage of rejections of the likelihood ratio test (LRT) between a positive stable frailty model and a proportional hazard model compared to the test for non-proportional hazards (ZPH), when the data are simulated without unobserved common risk and an increasing Weibull baseline hazard with shape $\alpha = 1$. The rows correspond to the total sample size $(300, 900, 1500)$ and the columns to the three main simulation scenarios: clustered failures, clustered failures where the observed covariate only varies between clusters, and recurrent events. β_1 indicates the strength of the time-dependent covariate effect.

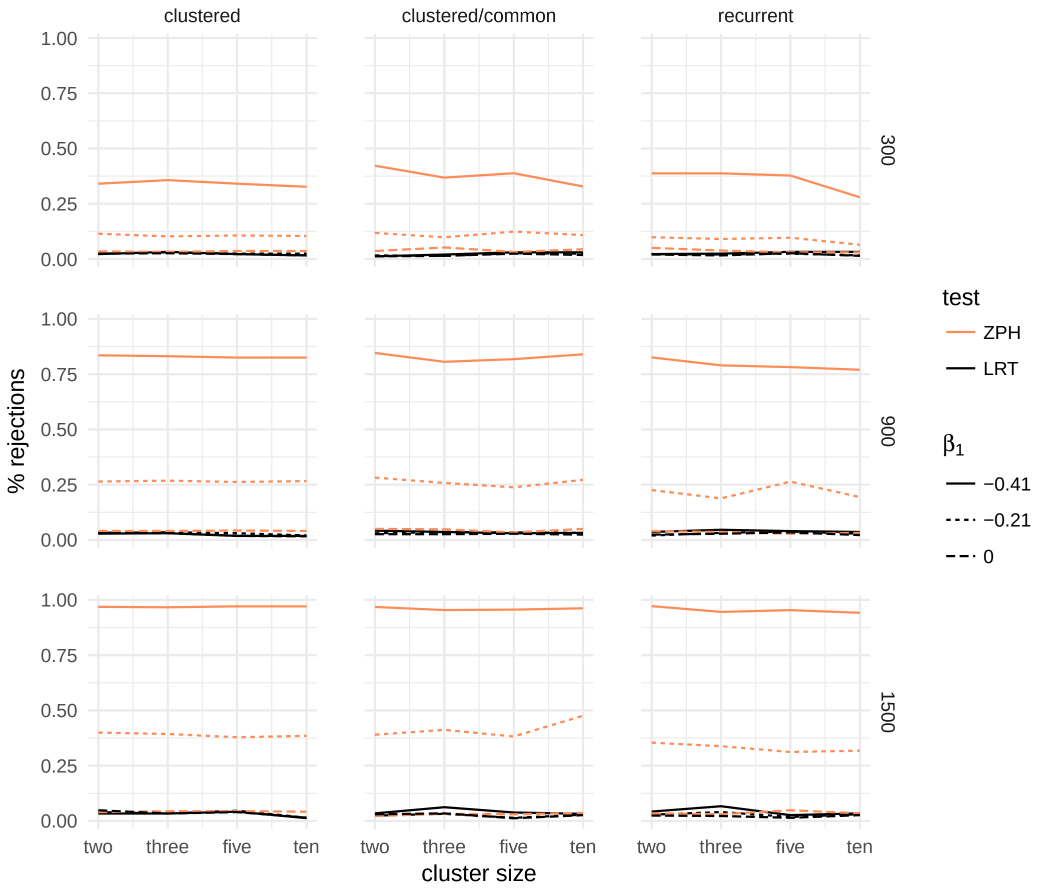

Figure S6: Percentage of rejections of the likelihood ratio test (LRT) between a positive stable frailty model and a proportional hazard model compared to the test for non-proportional hazards (ZPH), when the data are simulated without unobserved common risk and an increasing Weibull baseline hazard with shape $\alpha = 2$. The rows correspond to the total sample size $(300, 900, 1500)$ and the columns to the three main simulation scenarios: clustered failures, clustered failures where the observed covariate only varies between clusters, and recurrent events. β_1 indicates the strength of the time-dependent covariate effect.

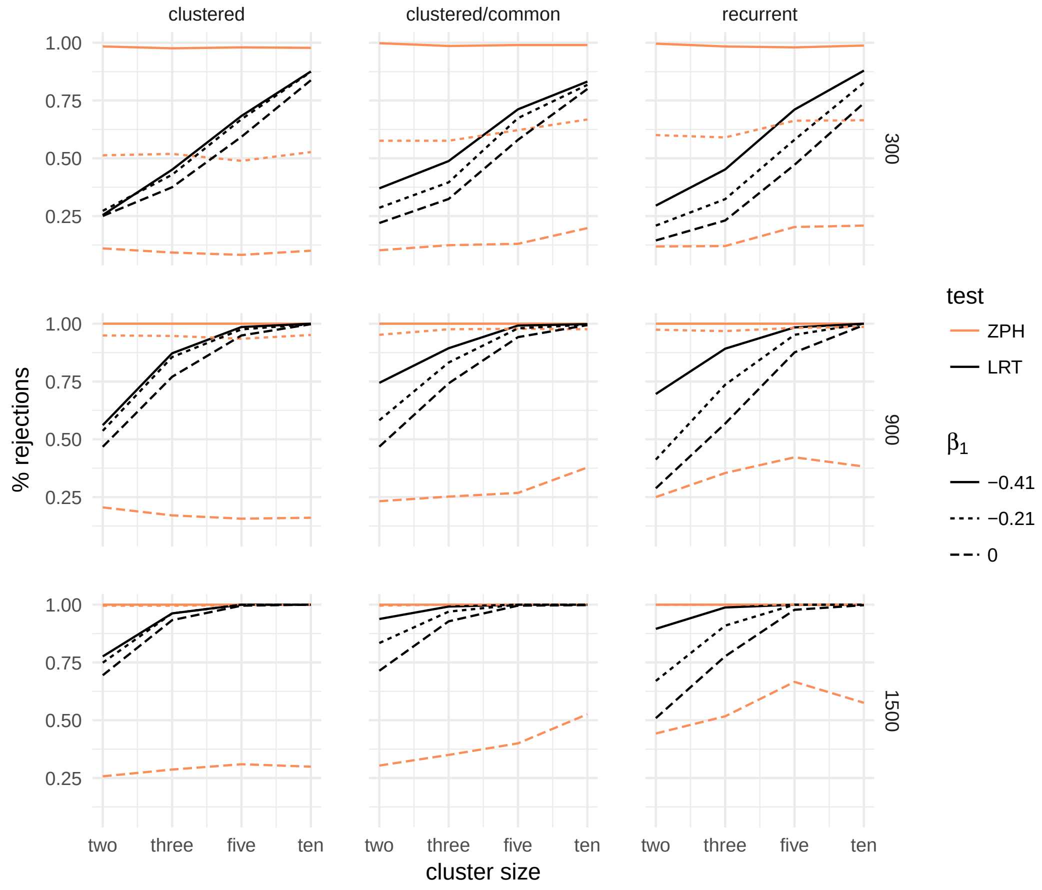

Figure S7: Percentage of rejections of the likelihood ratio test (LRT) between a positive stable frailty model and a proportional hazard model compared to the test for non-proportional hazards (ZPH), when the data are simulated with an unobserved common risk following a log-normal distribution with expectation 1 and variance 0.25 and an increasing Weibull baseline hazard with shape $\alpha = 1$. The rows correspond to the total sample size $(300, 900, 1500)$ and the columns to the three main simulation scenarios: clustered failures, clustered failures where the observed covariate only varies between clusters, and recurrent events. β_1 indicates the strength of the time-dependent covariate effect.

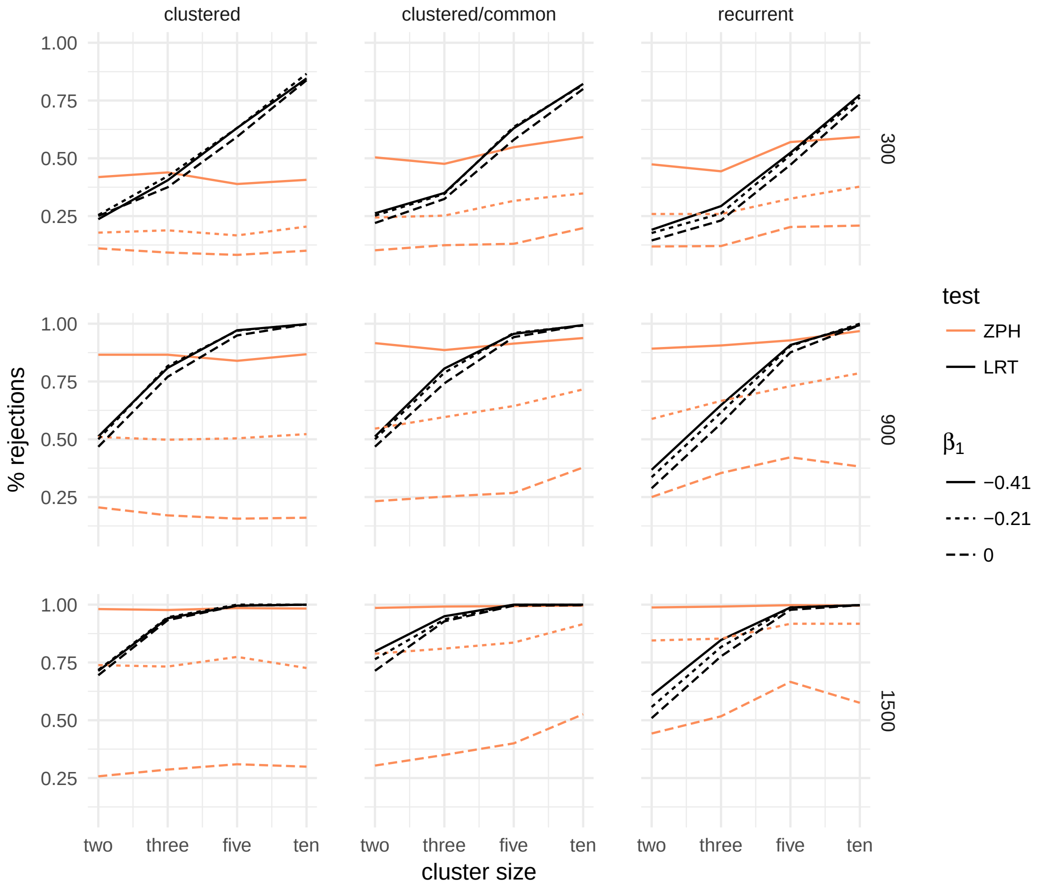

Figure S8: Percentage of rejections of the likelihood ratio test (LRT) between a positive stable frailty model and a proportional hazard model compared to the test for non-proportional hazards (ZPH), when the data are simulated with an unobserved common risk following a log-normal distribution with expectation 1 and variance 0.25 and an increasing Weibull baseline hazard with shape $\alpha = 2$. The rows correspond to the total sample size $(300, 900, 1500)$ and the columns to the three main simulation scenarios: clustered failures, clustered failures where the observed covariate only varies between clusters, and recurrent events. β_1 indicates the strength of the time-dependent covariate effect.

Likelihood Ratio Test - Inverse Gaussian

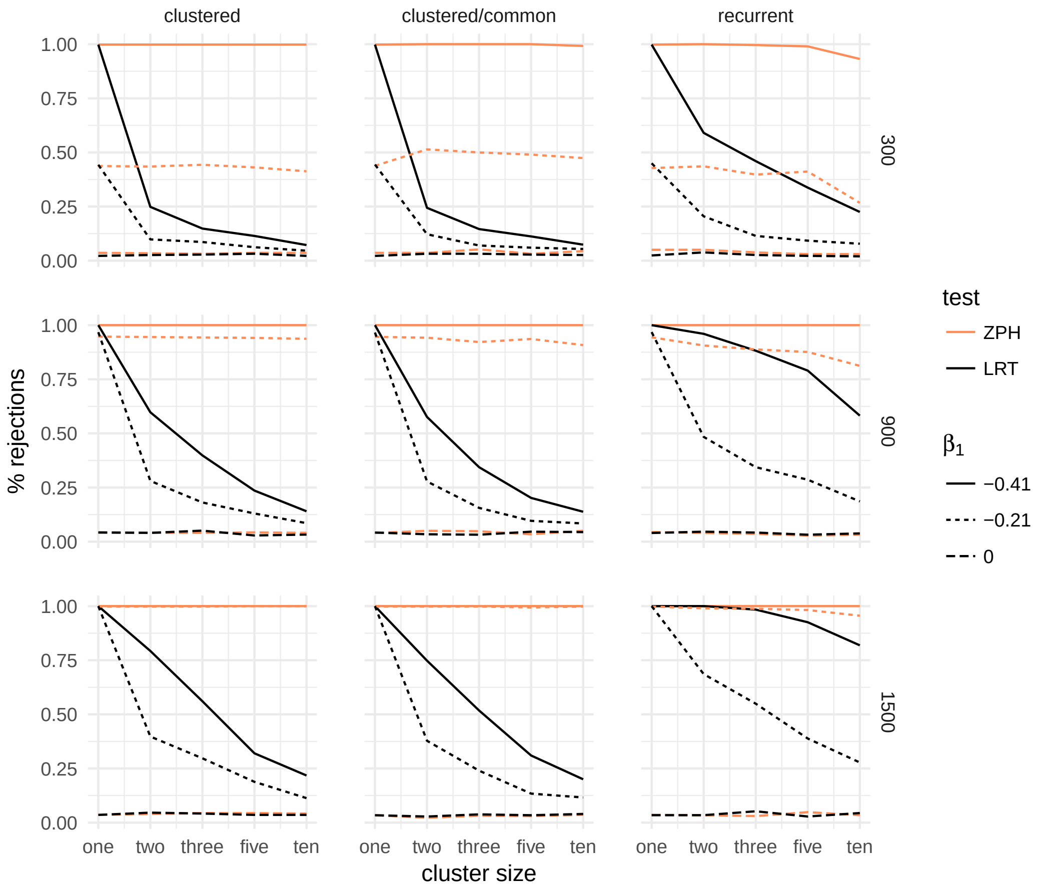

Figure S9: Percentage of rejections of the likelihood ratio test (LRT) between an inverse Gaussian frailty model and a proportional hazard model compared to the test for non-proportional hazards (ZPH), when the data are simulated without unobserved common risk and an increasing Weibull baseline hazard with shape $\alpha = 0.8$. The rows correspond to the total sample size $(300, 900, 1500)$ and the columns to the three main simulation scenarios: clustered failures, clustered failures where the observed covariate only varies between clusters, and recurrent events. β_1 indicates the strength of the time-dependent covariate effect.

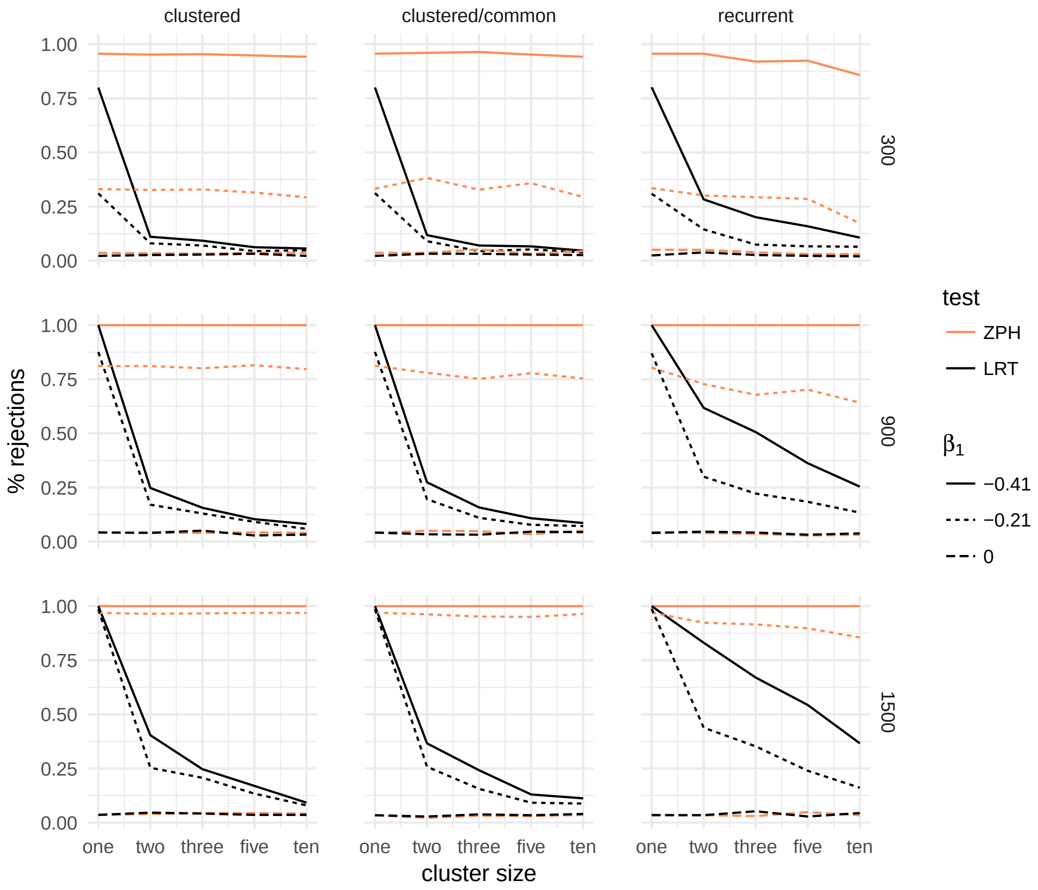

Figure S10: Percentage of rejections of the likelihood ratio test (LRT) between an inverse Gaussian frailty model and a proportional hazard model compared to the test for non-proportional hazards (ZPH), when the data are simulated without unobserved common risk and an increasing Weibull baseline hazard with shape $\alpha = 1$. The rows correspond to the total sample size $(300, 900, 1500)$ and the columns to the three main simulation scenarios: clustered failures, clustered failures where the observed covariate only varies between clusters, and recurrent events. β_1 indicates the strength of the time-dependent covariate effect.

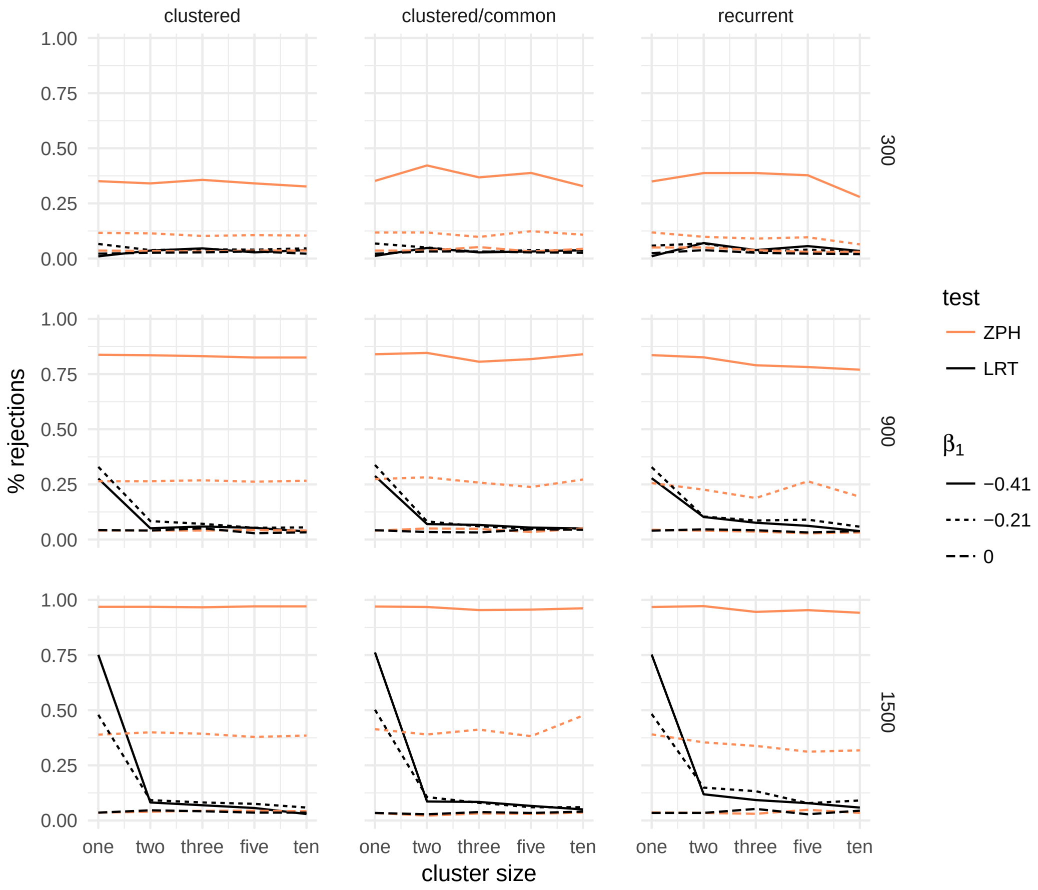

Figure S11: Percentage of rejections of the likelihood ratio test (LRT) between an inverse Gaussian frailty model and a proportional hazard model compared to the test for non-proportional hazards (ZPH), when the data are simulated without unobserved common risk and an increasing Weibull baseline hazard with shape $\alpha = 2$. The rows correspond to the total sample size $(300, 900, 1500)$ and the columns to the three main simulation scenarios: clustered failures, clustered failures where the observed covariate only varies between clusters, and recurrent events. β_1 indicates the strength of the time-dependent covariate effect.

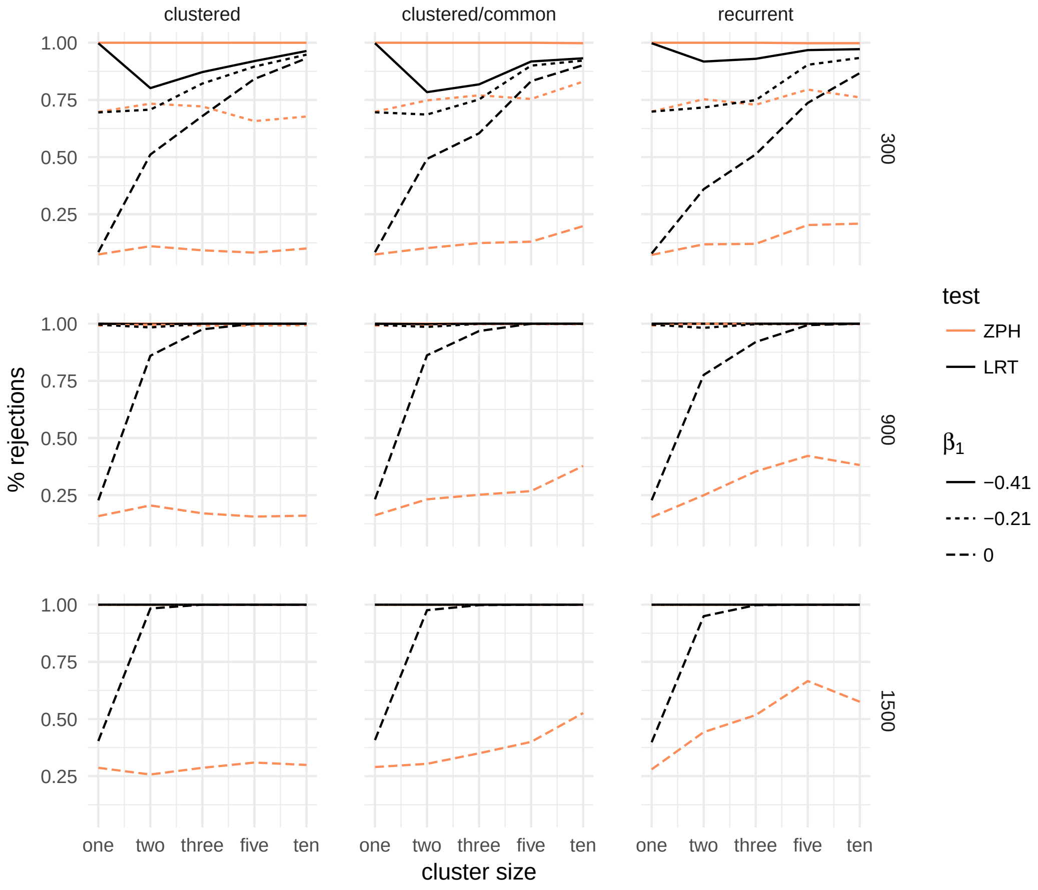

Figure S12: Percentage of rejections of the likelihood ratio test (LRT) between an inverse Gaussian frailty model and a proportional hazard model compared to the test for non-proportional hazards (ZPH), when the data are simulated with an unobserved common risk following a log-normal distribution with expectation 1 and variance 0.25 and an increasing Weibull baseline hazard with shape $\alpha = 1$. The rows correspond to the total sample size $(300, 900, 1500)$ and the columns to the three main simulation scenarios: clustered failures, clustered failures where the observed covariate only varies between clusters, and recurrent events. β_1 indicates the strength of the time-dependent covariate effect.

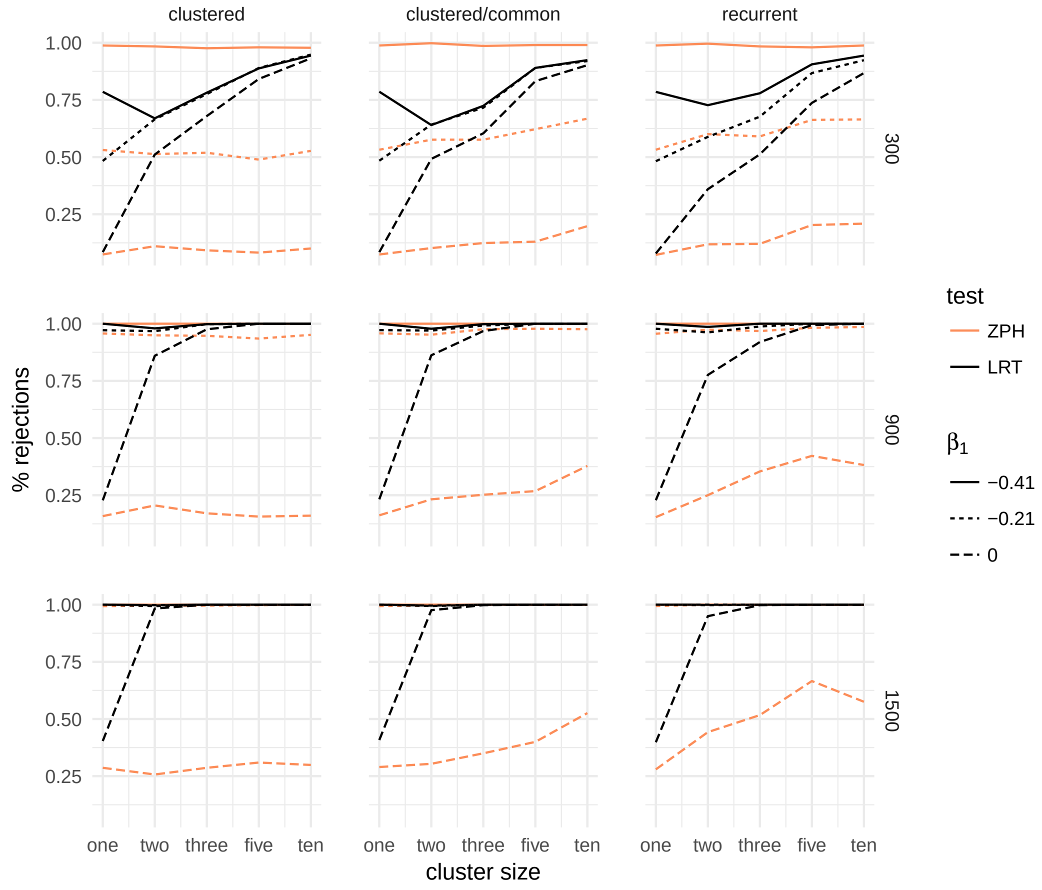

Figure S13: Percentage of rejections of the likelihood ratio test (LRT) between an inverse Gaussian frailty model and a proportional hazard model compared to the test for non-proportional hazards (ZPH), when the data are simulated with an unobserved common risk following a log-normal distribution with expectation 1 and variance 0.25 and an increasing Weibull baseline hazard with shape $\alpha = 1$. The rows correspond to the total sample size $(300, 900, 1500)$ and the columns to the three main simulation scenarios: clustered failures, clustered failures where the observed covariate only varies between clusters, and recurrent events. β_1 indicates the strength of the time-dependent covariate effect.

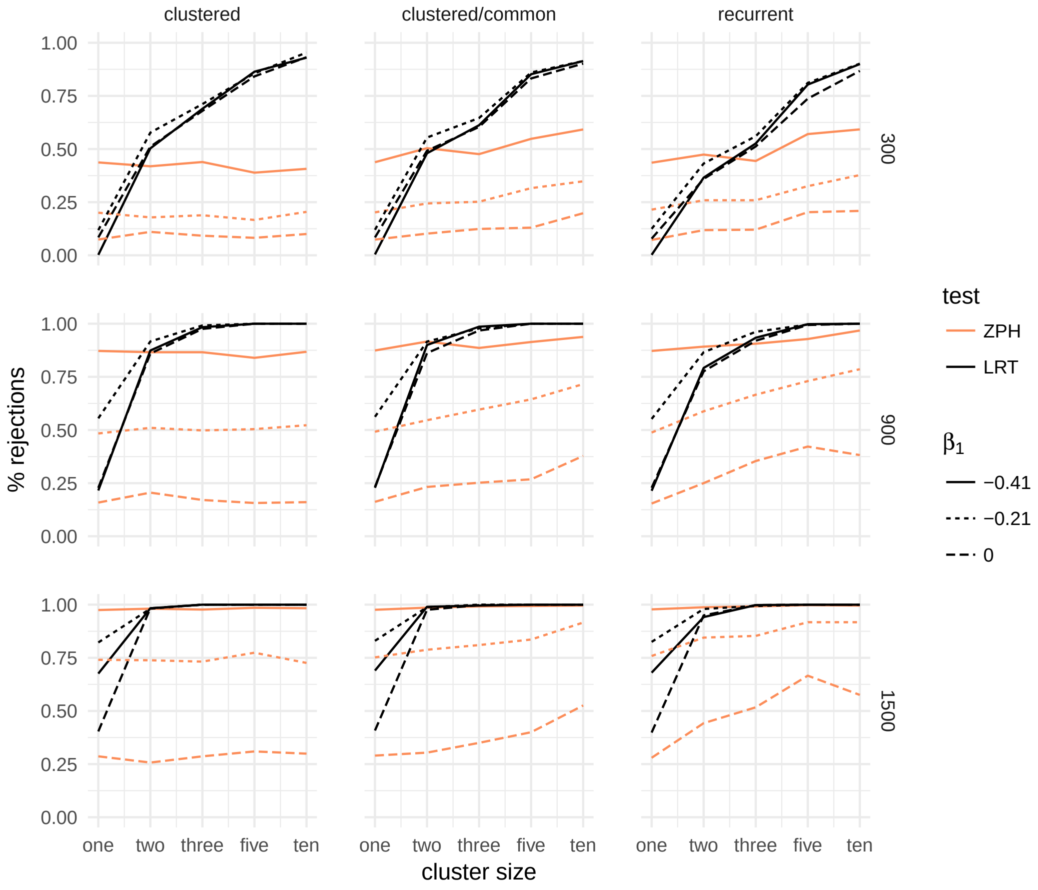

Figure S14: Percentage of rejections of the likelihood ratio test (LRT) between an inverse Gaussian frailty model and a proportional hazard model compared to the test for non-proportional hazards (ZPH), when the data are simulated with an unobserved common risk following a log-normal distribution with expectation 1 and variance 0.25 and an increasing Weibull baseline hazard with shape $\alpha = 1$. The rows correspond to the total sample size $(300, 900, 1500)$ and the columns to the three main simulation scenarios: clustered failures, clustered failures where the observed covariate only varies between clusters, and recurrent events. β_1 indicates the strength of the time-dependent covariate effect.
